# Characteristics Associated With High-Performing Pediatric Heart Transplant Centers in the United States From 2006 to 2015

**DOI:** 10.1001/jamanetworkopen.2020.23515

**Published:** 2020-11-02

**Authors:** Tajinder P. Singh, Mandeep R. Mehra, Kimberlee Gauvreau

**Affiliations:** 1 Department of Cardiology, Boston Children’s Hospital, Boston, Massachusetts; 2 Department of Pediatrics, Harvard Medical School, Boston, Massachusetts; 3 Heart and Vascular Center, Brigham and Women’s Hospital, Boston, Massachusetts; 4 Department of Medicine, Harvard Medical School, Boston, Massachusetts; 5 Department of Biostatistics, Harvard School of Public Health, Boston, Massachusetts

## Abstract

**Importance:**

Differences among pediatric transplant centers in long-term survival of pediatric recipients of heart transplants can be mostly explained by differences in 90-day mortality.

**Objective:**

To understand characteristics associated with high-performing pediatric HT centers by comparing key outcomes among centers stratified by 90-day risk-adjusted mortality.

**Design, Setting, and Participants:**

This retrospective cohort study included recipients of HT aged younger than 18 years in the US. Analyses included 44 US centers during 2006 to 2015 using the Organ Procurement and Transplant Network database. A risk model for 90-day mortality was developed using data from all recipients to estimate expected 90-day mortality and 90-day standardized mortality ratio (SMR; calculated as observed mortality divided by expected mortality) for each center. Centers were stratified into tertiles by SMR and compared for key outcomes. Data were analyzed from January to March 2020.

**Exposures:**

High-, medium-, and low-performing centers (SMR tertile).

**Main Outcomes and Measures:**

Posttransplant 90-day mortality across recipient risk spectrum and incidence of and mortality following early posttransplant complications.

**Results:**

Of 3211 children analyzed, 1016 (31.6%) were infants younger than 1 year and 1459 (45.4%) were girls. The median (interquartile range) age was 4 (0-12) years. Centers were stratified by SMR tertile, and SMR was 0 to 0.71 among 15 high-performing centers, 0.79 to 1.12 among 14 medium-performing centers, and 1.19 to 3.33 among 15 low-performing centers. High-performing centers had 90-day mortality of 0.8% (95% CI, 0.3%-1.8%) in children with low risk and expected mortality of 2.0%, 2.3% (95% CI, 0.6%-5.7%) in children with intermediate risk and expected mortality of 6.5%, and 16.7% (95% CI, 7.9%-29.3%) in children with high risk and expected mortality of 30.8%. Incidence of acute rejection during transplant hospitalization was 10.3% at high-performing centers, 10.3% at medium-performing centers, and 9.7% at low-performing centers (*P* for trend = .68), and incidence of post-HT kidney failure requiring dialysis was 4.1% at high-performing centers, 5.2% at medium-performing centers, and 8.5% at low-performing centers (*P* for trend = .001). Ninety-day mortality was significantly lower at high-performing centers among children treated for rejection (high-performing: 2.0%; medium-performing: 6.9%; low-performing: 11.7%; *P* for trend = .006) and among recipients receiving dialysis for post-HT kidney failure (high-performing: 17.5%; medium-performing: 39.4%; low-performing: 47.6%; *P* for trend < .001).

**Conclusions and Relevance:**

This cohort study found that high-performing pediatric HT centers had lower 90-day mortality across the recipient risk spectrum and lower mortality among recipients who develop rejection or post-HT kidney failure during transplant hospitalization. These findings suggest presence of superior processes and systems of care at high-performing pediatric HT centers.

## Key Points

**Question:**

What are the characteristics associated with high-performing pediatric heart transplant (HT) centers?

**Findings:**

In this cohort study of 3211 recipients of HT younger than 18 years at 44 pediatric HT centers in the US during 2006 to 2015, the top one-third of centers by performance had significantly lower 90-day mortality across recipient risk spectrum and significantly lower 90-day mortality in recipients who developed rejection or posttransplant kidney failure during transplant hospitalization.

**Meaning:**

These findings suggest the presence of superior processes and systems of care at high-performing pediatric HT centers and may provide insights for designing quality improvement initiatives at low-performing centers.

## Teaser

This cohort study examines characteristics of high-performing pediatric heart transplant centers in the United States.

## Introduction

Heart transplant (HT) in children younger than 18 years is currently offered at more than 60 centers in the United States, and these centers perform 400 to 450 transplants per year.^1^ Although some of these are adult centers performing occasional HT in older children or teenagers, most are low-volume pediatric HT centers performing fewer than 5 transplants annually. With such a large number of low-volume centers and overall 1-year survival that exceeds 90%, discerning outcome differences among centers adjusted for case-mix is difficult. We have previously described risk modeling for early mortality after HT in children using baseline characteristics at HT.^2,3^ Such models, when generated using the entire cohort, can be used to assess center case-mix, assessed as expected mortality, and center performance, assessed as standardized mortality ratio (SMR; the ratio of observed to expected mortality), with lower SMR in higher-performing centers.^4^ Using this approach, our previous work has demonstrated that most of the outcome differences between centers materialize within the first 90 days after transplant.^4^ High-performing centers have lower risk-adjusted 90-day mortality and maintain their performance advantage over other centers on longer-term follow-up.

Previous studies have often focused on center volume as the key characteristic associated with pediatric HT outcomes.^5,6^ While countries with national health care systems usually offer HT at a small number of large-volume centers, many pediatric centers in the US are low-volume centers. Understanding signature characteristics of high-performing centers may guide quality improvement (QI) initiatives at low-performing centers that may help improve HT outcomes at these centers and overall.^7^ We hypothesized that high-performing pediatric HT centers have significantly better 90-day outcomes in higher-risk recipients.

The specific aims of this study were to compare high- and low-performing US pediatric HT centers for 90-day posttransplant mortality across recipient risk spectrum, 90-day cause-specific mortality, and incidence of rejection and posttransplant kidney failure requiring dialysis during the transplant hospitalization and 90-day posttransplant mortality among recipients with these complications.

## Methods

### Study Setting and Participants

We identified all children younger than 18 years in the Organ Procurement and Transplant Network (OPTN) database who received primary HT in the US between January 1, 2006, and December 31, 2015, at active HT centers, defined as centers that performed at least 1 de novo pediatric HT in 2014 to 2015. These data are provided as deidentified data by the United Network for Organ Sharing (UNOS) to the investigators and are considered institutional review board and informed consent exempt under Federal Regulation 45 CFR 46.101(b). The Health Resources and Services Administration of the Department of Health and Human Services provides oversight to the activities of UNOS. Posttransplant follow-up was available in all participants until March 31, 2016. This study is reported following the Strengthening the Reporting of Observational Studies in Epidemiology (STROBE) reporting guideline.

Children who received heart retransplantation or multiorgan transplantation were excluded. We also excluded centers if they had not performed an HT in a child younger than 10 years (to exclude adult centers), were inactive for 5 years consecutively, or had performed a total of fewer than 10 HT procedures during the 10-year study period. The OPTN database includes baseline demographic and clinical information at HT in all recipients in the US submitted by transplant centers. These data are supplemented with death data from the Social Security master death file.

### Study Design and Variables

This was a retrospective cohort study. The data were analyzed during January to March 2020. The primary outcome was 90-day posttransplant mortality across recipient risk spectrum. The exposure was high-, medium-, and low-performing centers, stratified using 90-day SMR for each center (SMR tertiles).

Demographic and clinical variables were defined at transplant. Race/ethnicity was recorded as reported by the center and analyzed as White (ie, non-Hispanic White), Black (ie, non-Hispanic Black), Hispanic, or other. Kidney function was analyzed as estimated glomerular filtration rate (eGFR, in mL/min/1.73 m^2^) using serum creatinine and the modified Schwartz equation.^8^ For children aged 1 year or older, normal kidney function was defined as eGFR greater than 60; moderate dysfunction, eGFR 30 to 60; and severe dysfunction, eGFR less than 30 or dialysis support. For infants younger than 1 year, normal kidney function was defined as eGFR greater than 40; moderate dysfunction, eGFR 20 to 40; and severe dysfunction, eGFR less than 20 or dialysis support.^9^

There were no missing data for age, sex, race/ethnicity, cardiac diagnosis, blood type, hemodynamic support (ie, inotrope support, ventilator, and type of mechanical support), health insurance (ie, Medicaid), dialysis, and the dates of transplant, death, or retransplant. For children with missing values of serum creatinine or bilirubin, we used multiple imputation to impute their eGFR and serum bilirubin using clinical variables at transplant; 10 imputations were used for each missing value.^10^

### Statistical Analysis

Baseline recipient characteristics are presented as median (interquartile range [IQR]) or number (percentage). Expected 90-day mortality (ie, risk of 90-day mortality) for each patient was assessed using a risk model. This model was developed using the entire study cohort and considered baseline recipient characteristics at HT and year of transplant. We used logistic regression with forward selection, retaining variables significant at the .10 level based on a likelihood ratio test. Model discrimination was assessed using the area under the receiver operating characteristic curve (ie, *C* statistic) and calibration. The model was internally validated using bootstrapping with 100 random samples and 3211 patients per sample with replacement. Model coefficients were used to estimate the probability of death within 90 days for each recipient, the expected 90-day mortality for each center (ie, case-mix) and 90-day SMR (ie, performance) for each center. Centers were stratified as high-, medium-, or low-performing based on SMR tertiles. Baseline characteristics of recipients at these centers were compared using the χ^2^ test for categorical variables and the Kruskal-Wallis test for continuous variables. Observed (with 95% CIs) vs model-estimated expected mortality was assessed in the 3 SMR groups across the recipient risk spectrum. The groups were also compared for cause-specific 90-day mortality, incidence of treated rejection and posttransplant kidney failure, and mortality within 90 days after HT following these complications using tests for trend performed using logistic regression, adjusting the SEs of model coefficients to account for the clustering of patients within centers.

Data were analyzed using SAS statistical software version 9.4 (SAS Institute) and Stata version 15 (StataCorp). All statistical tests were 2-sided, and *P* < .05 was considered statistically significant.

## Results

During the 10-year study period, 3495 children younger than 18 years underwent their first HT at 86 US transplant centers. Of these, 31 centers were inactive during 2014 and 2015, 6 centers performed fewer than 10 HT procedures during the study period, and 5 centers were inactive during 5 consecutive years. These 42 centers (and 284 recipients of HT at these centers) were excluded from further analysis. The remaining 44 centers with 3211 recipients formed the study cohort. Of these, 1016 (31.6%) were infants younger than 1 year, and 1459 (45.4%) were girls. The median (interquartile range) age was 4 (0-12 years. Cardiac diagnosis was dilated cardiomyopathy in 1387 recipients (43.2%), nondilated cardiomyopathy in 271 recipients (8.4%), and congenital heart disease in 1429 recipients (44.5%). At the time of HT, 570 children (17.8%) were supported on a ventilator, 763 children (23.8%) were supported on a mechanical circulatory support, and 80 children (2.5%) were receiving dialysis.

The 44 study centers performed a median (IQR) of 59 (35-114) transplants during the 10-year period. The median (IQR) annual transplant volume among centers was 5.9 (3.5-11.4) transplants per year, with a range of 1.0 to 19.2 transplants.

### Risk-Standardization for 90-Day Mortality

**Figure 1.**
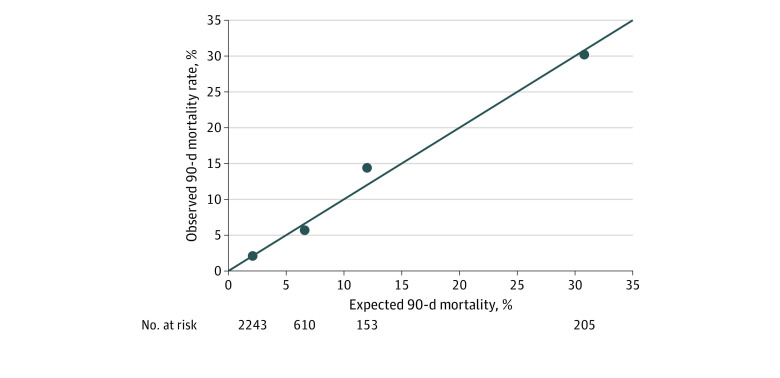
Observed vs Expected Posttransplant 90-Day Mortality Using the Risk Model in All Recipients of Heart Transplants The line indicates the line of identity; dots, observed vs the expected mortality within the expected mortality ranges of less than 5%, 5% to 9.9%, 10% to 14.9%, and 15% or greater.

The risk model for 90-day posttransplant mortality (Table 1) had an excellent ability to discriminate children who died from those who did not (*C* statistic, 0.80) and was well calibrated. Figure 1 illustrates estimated vs observed mortality in study children with expected mortality of less than 5% (ie, low risk), 5% to 9.9%, 10% to 14.9%, and greater than 15% (ie, high risk). On internal validation by bootstrapping, the *C* statistic ranged from 0.76 to 0.84 in repeated samples (optimism-corrected mean 0.81; 95% CI, 0.80-0.81). Using this model, the risk of death within 90 days of HT ranged from 0.5% to 82.0% among study children (median [IQR] risk, 2.7% [1.4%-5.5%]). Expected 90-day mortality at study centers ranged from 2.1% to 12.4% (median [IQR] risk, 5.1% [3.4%-5.9%]).

**Table 1.** Multivariable Risk Model for Mortality Within 90 Days of Heart Transplant

Characteristic	Coefficient	Odds ratio (95% CI)^a^
Age at transplant, y		
<1	0.9928	2.70 (1.59-4.59)
1-10	0.3022	1.35 (0.81-2.25)
11-17	NA	1 [Reference]
Diagnosis		
Dilated CMP	NA	1 [Reference]
Non-dilated CMP	0.9409	2.56 (1.20-5.48)
CHD, repaired	1.3901	4.02 (2.54-6.34)
CHD, unrepaired	1.0464	2.85 (1.42-5.71)
Other	0.0134	1.01 (0.29-3.49)
Ventilator	0.3936	1.48 (1.00-2.19)
Mechanical support		
None	NA	1 [Reference]
ECMO	1.5365	4.65 (2.93-7.37)
BIVAD	1.1620	3.20 (1.56-6.53)
LVAD	0.2185	1.24 (0.63-2.46)
Baseline kidney dysfunction		
None	NA	1 [Reference]
Moderate	0.3967	1.49 (0.97-2.29)
Severe	1.5377	4.65 (2.78-7.78)
Bilirubin, mg/dL		
<0.6	NA	1 [Reference]
0.6-1.9	0.4096	1.51 (1.01-2.25)
≥2.0	0.9169	2.50 (1.57-3.99)
Intercept	–5.2809	NA

Abbreviations: BIVAD, biventricular assist device; CMP, cardiomyopathy; CHD, congenital heart disease; ECMO, extracorporeal membrane oxygenation; LVAD, left ventricular assist device; NA, not applicable.

SI conversion factor: To convert bilirubin to micromoles per liter, multiply by 17.104.

^a^ Calculated as *P* = (*X*/*X*+1) where *X* indicates exp (intercept + coefficient for each variable as it applies to the patient).

### Center Stratification by 90-Day Performance

Observed 90-day mortality was 5.2% overall and ranged from 0% to 23.1% (median [IQR] risk, 4.9% [3.1%-6.7%]) among centers; SMR for 90-day mortality ranged from 0 to 3.33 (median [IQR], 0.92 [0.59-1.30]). Centers were stratified based on 90-day SMR into 3 approximately equal-sized groups of 15 high-performing centers (SMR range, 0 to 0.71), 14 medium-performing centers (SMR range, 0.79 to 1.12) and 15 low-performing centers (SMR range, 1.19 to 3.33). Overall HT volume among the 3 groups was 973 transplants in the high-performing centers, 1268 transplants at medium-performing centers, and 970 transplants at low-performing centers. Baseline characteristics of study children at centers stratified by 90-day SMR are shown in Table 2. Although the 3 groups were significantly different in distribution of several variables, there were no significant trends across high- to medium- to low-performing centers for these variables.

**Table 2.** Baseline Characteristics of Recipients of Heart Transplants at High-, Medium-, and Low-Performing Centers

Variable	Patients by center, No. (%)			*P* value
	High-performing (n = 973)	Medium-performing (n = 1268)	Low-performing (n = 970)	
Age at transplant, y				
<1	286 (29.4)	419 (33.0)	311 (32.1)	.18
1-10	377 (38.8)	493 (38.9)	356 (36.7)	
11-17	310 (31.9)	356 (28.1)	303 (31.2)	
Girls	432 (44.4)	584 (46.1)	443 (45.7)	.73
Diagnosis				
Dilated CMP	447 (45.9)	527 (41.6)	413 (42.6)	.09
Nondilated CMP	85 (8.7)	100 (7.9)	86 (8.9)	
CHD, repaired	362 (37.2)	507 (40.0)	370 (38.1)	
CHD, unrepaired	39 (4.0)	84 (6.6)	67 (6.9)	
Other	40 (4.1)	50 (3.9)	34 (3.5)	
Blood type				
A	327 (33.6)	477 (37.6)	348 (35.9)	.13
AB	37 (3.8)	55 (4.3)	42 (4.3)	
B	140 (14.4)	149 (11.8)	148 (15.3)	
O	469 (48.2)	587 (46.3)	432 (44.5)	
Race/Ethnicity				
White	456 (46.9)	778 (61.4)	516 (53.2)	<.001
Black	217 (22.3)	242 (19.1)	196 (20.2)	
Hispanic	221 (22.7)	160 (12.6)	195 (20.1)	
Other	79 (8.1)	88 (6.9)	63 (6.4)	
Inotropes	485 (49.9)	655 (51.7)	478 (49.3)	.49
Ventilator	160 (16.4)	270 (21.3)	140 (14.4)	<.001
Mechanical support				
ECMO	45 (4.6)	85 (6.7)	48 (5.0)	<.001
BIVAD	36 (3.7)	85 (6.7)	29 (3.0)	
LVAD	151 (15.5)	144 (11.4)	140 (14.4)	
Dialysis	20 (2.1)	39 (3.1)	21 (2.2)	.24
Kidney dysfunction				
Moderate	257 (26.4)	279 (22.0)	242 (25.0)	.01
Severe	30 (3.1)	69 (5.4)	37 (3.8)	
Bilirubin, mg/dL				
<0.6	413 (42.4)	591 (46.6)	425 (43.8)	.23
0.6-1.9	429 (44.1)	532 (42.0)	413 (42.5)	
≥2.0	131 (13.5)	145 (11.4)	132 (13.6)	
PRA (%)				
≤10	759 (78.0)	1038 (81.9)	737 (76.0)	<.001
>10, ≤25	72 (7.4)	61 (4.8)	51 (5.3)	
>25	142 (14.6)	169 (13.3)	182 (18.8)	
Medicaid insurance	440 (45.2)	595 (46.9)	351 (36.2)	<.001
Year of transplant				
2006-2008	246 (25.3)	319 (25.2)	283 (29.2)	.01
2009-2011	266 (27.3)	396 (31.2)	297 (30.6)	
2012-2015	461 (47.4)	553 (43.6)	390 (40.2)	

Abbreviations: BIVAD, bi-ventricular assist device; CHD, congenital heart disease; CMP, cardiomyopathy; ECMO, extra-corporeal membrane oxygenation; LVAD, left ventricular assist device; PRA, panel reactive antibody.

SI conversion factor: To convert bilirubin to micromoles per liter, multiply by 17.104.

Expected 90-day mortality at high-performing centers was 4.8%, at medium-performing centers was 5.8%, and at low-performing centers was 5.0%. Observed 90-day mortality was 2.4% at high-performing centers, 5.2% at medium-performing centers, and 8.1% at low-performing centers. Median (IQR) annual HT volume was 4.4 (1.9-12.5) transplants/year at high-performing centers, 9.7 (3.5-13.8) transplants/year at medium-performing centers, and 5.8 (4.0-9.1) transplants/year at low-performing centers (*P* = .25). Seven of 15 high-performing centers (all with annual volume <5.1 transplants/year) had a low-severity case-mix, with expected 90-day mortality between 2.1% and 3.3%. There was no trend in distribution of donor variables (ie, ischemic time, percentage of donors using multiple inotropes or with left ventricular ejection fraction <0.45, donor:recipient weight ratio <0.8, or percentage of donors with >20% body surface area difference with the recipient) among centers stratified by performance (eTable in the Supplement).

### Mortality at Centers Stratified by 90-Day Performance

**Figure 2.**
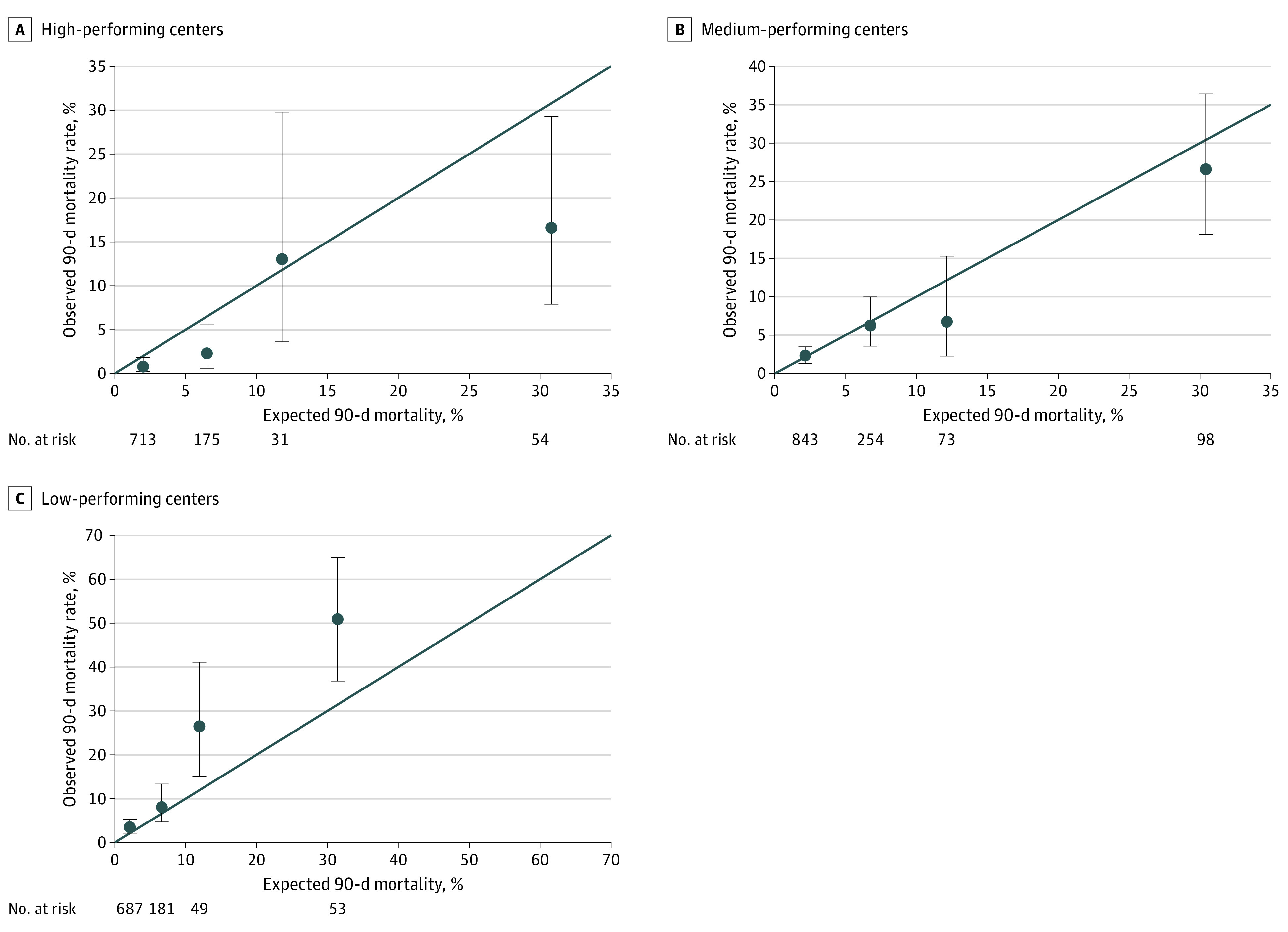
Observed vs Expected Posttransplant 90-Day Mortality The line indicates the line of identity; dots, observed vs the expected mortality within the expected mortality ranges of less than 5%, 5% to 9.9%, 10% to 14.9%, and 15% or greater; and error bars, 95% CIs for observed mortality.

Figure 2 illustrates observed 90-day mortality vs expected mortality assessed using the risk model across the recipient risk spectrum at high-, medium-, and low-performing centers. The observed 90-day mortality at high-performing centers was 0.8% (95% CI, 0.3%-1.8%) in children with low risk and expected 90-day mortality of 2.0%, 2.3% (95% CI, 0.6%-5.7%) in children with intermediate risk and expected mortality of 6.5%, and 16.7% (95% CI, 7.9%-29.3%) in children with high risk and expected mortality of 30.8% (Figure 2A). The observed 90-day mortality was as expected in medium-performing centers across the recipient risk spectrum (Figure 2B). The observed 90-day mortality at low-performing centers was 26.5% (95% CI, 14.9%-41.1%) in children with intermediate risk and expected mortality of 11.9% and 50.9% (95% CI, 36.8%-64.9%) in children with high risk and expected mortality of 31.4% (Figure 2C). Comparison of cause-specific mortality, as defined in the database, showed lower mortality associated with graft failure, stroke, multiorgan failure, infection, and hemorrhage deaths in high-performing centers (Table 3).

**Table 3.** Cause-Specific Mortality at High-, Medium-, and Low-Performing Centers

Cause of death	Deaths by center, No. (%)			*P* for trend
	High performing (n = 973)	Medium performing (n = 1268)	Low performing (n = 970)	
Graft failure	6 (0.6)	22 (1.7)	22 (2.3)	<.001
Infection	0	5 (0.4)	5 (0.5)	.02
Cardiovascular	2 (0.2)	8 (0.6)	4 (0.4)	.32
Pulmonary	4 (0.4)	4 (0.3)	2 (0.2)	.38
Cerebrovascular	3 (0.3)	10 (0.8)	11 (1.1)	.009
Hemorrhage	2 (0.2)	4 (0.3)	9 (0.9)	.02
Malignant neoplasm	0	0	0	NA
Multiorgan failure	3 (0.3)	8 (0.6)	19 (2.0)	<.001
Other	2 (0.2)	3 (0.2)	5 (0.5)	.36
Unknown or not reported	1 (0.1)	2 (0.2)	2 (0.2)	.50

Abbreviation: NA, not applicable.

### Early Posttransplant Complications and Associated Mortality

Acute rejection was treated prior to posttransplant discharge in 100 recipients (10.3%) at high-performing centers, 131 recipients (10.3%) at medium-performing centers, and 94 recipients (9.7%) at low-performing centers (*P* for trend = .68). Among children treated for acute rejection, 90-day mortality was significantly lower at high-performing centers (2 recipients [2.0%]) than at medium-performing (9 recipients [6.9%]) and low-performing (11 recipients [11.7%]) centers (*P* for trend = .006).

Overall, 188 recipients (5.8%) required posttransplant dialysis during transplant hospitalization. Of these, 42 recipients were already receiving dialysis at transplant, whereas 146 recipients developed de novo kidney failure. While the distribution among groups was similar at transplant, posttransplant kidney failure was observed in 40 recipients (4.1%) at high-performing centers, 66 recipients (5.2%) at medium-performing centers, and 82 recipients (8.5%) at low-performing centers (*P* for trend = .001). Among children who required posttransplant dialysis, 90-day mortality was significantly lower at high- performing centers (7 recipients [17.5%]) compared with medium-performing (26 recipients [39.4%]) and low-performing (39 recipients [47.6%]) centers (*P* for trend < .001).

## Discussion

We have previously reported that center differences in long-term survival of pediatric recipients of HT can be explained mostly by differences in center performance (risk-adjusted mortality) within the first 90 days.^4^ In this study, we stratified US centers based on their 90-day SMR over a 10-year period into equal number of high-, medium-, and low-performing centers to further understand what high-performing centers did better. We found that in general, there were no significant differences in transplant volume or case-mix among the 3 groups. High-performing centers had statistically significantly lower-than-expected 90-day mortality across the recipient risk spectrum and had lower cause-specific mortality associated with most causes. Their recipients were less likely to develop kidney failure requiring dialysis prior to hospital discharge and were more likely to survive if they did. Although the incidence of treated rejection was similar among the 3 groups, 90-day mortality among those who developed rejection was lower at high-performing centers. Thus, patients were less likely to die at high-performing centers after developing kidney failure or rejection. These findings suggest presence of superior patient care processes and systems for managing HT at high-performing centers.

Several studies have reported worse posttransplant outcomes at low-volume HT centers.^5,6,11,12^ There was no statistically significant difference in distribution of transplant volume among the 3 groups in this current study, and half of high-performing centers were low-volume centers. The low expected mortality at these low-volume centers suggests that these centers limited their recipients to patients with low risk. The larger-volume high-performing centers, in contrast, accepted and transplanted many recipients with high risk with better-than-expected outcomes in such patients. Although these findings of volume vs case-mix within the stratum of high-performers may be somewhat intuitive, they illustrate that high-performing centers, irrespective of volume, were better at selecting and transplanting patients appropriate for their center’s expertise, systems, and resources at the time. The fact that institutional factors other than volume play an important role has also been reported for adult recipients, where center volume explained only 16.7% of center variability in 1-year post-HT mortality among US centers.^7^

A similar incidence of rejection was seen among the 3 groups and is not surprising, considering the availability and adoption of modern immune suppression at most centers.^13^ Early post-HT kidney failure requiring dialysis has been previously reported in 6% of pediatric recipients and is associated with pretransplant risk factors of patient complexity and severity of illness.^14^ Despite a similar case-mix and frequency of pretransplant kidney failure among groups, the incidence of posttransplant kidney failure was significantly lower at high-performing centers. However, 90-day mortality among recipients who developed rejection or kidney failure during transplant hospitalization was significantly lower at high-performing centers compared with medium- and low-performing centers. These comparative results have parallels in surgical literature, in which the term *failure to rescue* has been used to describe in-hospital death after adverse events, such as postsurgical complications.^15^ There is extensive discussion regarding this phenomenon in critical care and postsurgical literature, with focus on institutional quality of care.^16,17^ Whether or not one considers posttransplant deaths that follow rejection or acute kidney failure as failure to rescue, the systems of care at high-performing centers that allowed them to have much better outcomes in such patients could act as models for improving care at lower-performing centers.

We used the study cohort for developing the risk model rather than using a previously published model because our primary goal was risk adjustment among centers for severity of case-mix. Risk models perform best within the cohort in which they are developed, and the performance metric of the model was as good as in any previously reported model. We also chose the entire cohort to develop the model (rather than two-thirds or three-fourths of the cohort) to be able to use the full information for developing model coefficients.^18^

### Study Implications

Such center comparisons are potentially useful for developing QI initiatives at low-performing centers. Most QI initiatives in medicine are targeted to improve processes, with the ultimate goal to improve outcomes. Because early survival after HT is excellent in the current era, it is difficult to design and illustrate the effectiveness of any QI initiative targeting mortality in a reasonable time period. This is particularly true at pediatric HT centers, where a single death may dramatically reduce a center’s SMR with little chance of recovery until the event is no longer counted by the regulatory agencies. Our findings provide a potential roadmap for centers seeking to improve their early posttransplant outcomes through QI initiatives that focus on improving processes by benchmarking the observed-to-expected outcomes reported in this analysis, including the identification of the principle complications of acute rejection and kidney failure. An example may be for a transplant team to quantify the risk of early posttransplant mortality using a published risk model in candidates for HT instead of labeling such patients as low or high risk based on the team’s perception. When considered in the context of posttransplant outcomes of patients with similar risk at their center in recent years, specific interventions, ranging from early engagement of specialists, referral to a different center or waiting to list until the patient risk can be lowered, that may improve the likelihood of patient survival may be considered. Another example would be to mandate a systematic evaluation or root-cause analysis of center processes and systems following one of the well-defined early posttransplant events, such as severe rejection, kidney failure, or death, to seek changes in existing processes that may prevent such events in the future.

### Limitations

This study has several limitations. First, this was a retrospective study using registry data with inherent limitations of such data. However, submission of these data to UNOS by centers is required, the data are periodically audited by UNOS, and the data used for the current analysis are identical to those used by the regulatory bodies to generate center-specific reports. Second, we excluded centers performing pediatric HT in children on a rare basis, and our findings may not be generalizable to such centers. Third, the OPTN data do not provide information on systems, processes, and clinical practices that allowed high-performing centers to achieve superior outcomes. Understanding these will require studies using different data sets or a prospective design.

## Conclusions

This cohort study found that high-performing pediatric HT centers had superior outcomes across recipient risk spectrum, lower cause-specific mortality associated with most causes, a lower incidence of acute kidney failure following transplant, and a higher rate of recovery following serious complications, such as allograft rejection and kidney failure. These findings suggest the presence of superior processes and systems of care for recent recipients of HT at these centers and may provide insights for designing QI initiatives at low-performing centers.

## Notes

Supplement. **eTable.** Distribution of Donor Variables in High-, Medium-, and Low-Performing Centers
