# Supplementary material for: Characteristics Associated With High-Performing Pediatric Heart Transplant Centers in the United States From 2006 to 2015
Source: JAMA Netw Open. 2020 Nov 2;3(11):e2023515. doi: 10.1001/jamanetworkopen.2020.23515 (PMC7607438; doi:10.1001/jamanetworkopen.2020.23515)
Supplement: Supplement. — eTable. Distribution of Donor Variables in High-, Medium-, and Low-Performing Centers [file jamanetwopen-e2023515-s001.pdf]

Supplemental Online Content

Singh TP, Mehra MR, Gauvreau K. Characteristics associated with high-performing pediatric heart transplant centers in the United States from 2006 to 2015. *JAMA Netw Open*. 2020;3(11):e2023515. doi:10.1001/jamanetworkopen.2020.23515

eTable. Distribution of Donor Variables in High-, Medium-, and Low-Performing Centers

This supplemental material has been provided by the authors to give readers additional information about their work.

© 2020 Singh TP et al. *JAMA Network Open.*

eTable. Distribution of Donor Variables in High-, Medium-, and Low-Performing Centers

	**High-Performing** (n=973)	**Medium-Performing** (n=1268)	**Low-Performing** (n=970)
**Donor Age** (years)			
< 1	206 (21.2%)	354 (27.9%)	250 (25.8%)
1 to 10	399 (41.0%)	506 (39.9%)	385 (39.7%)
≥ 11	368 (37.8%)	408 (32.2%)	335 (34.5%)
**Donor Age** (years)	5 [1, 15]	4 [0, 14]	4 [0, 15]
**Ischemic Time** (hours)			
≤ 3.5	488 (50.2%)	702 (55.4%)	453 (46.7%)
> 3.5	454 (46.7%)	549 (43.3%)	494 (50.9%)
Not reported	31 (3.2%)	17 (1.3%)	23 (2.4%)
**Ischemic Time** (hours)	3.6 [2.9, 4.3]	3.5 [2.9, 4.0]	3.6 [3.0, 4.3]
**On Multiple Inotropes**	13 (1.3%)	18 (1.4%)	14 (1.4%)
**LV Ejection Fraction < 45%**	23 (2.4%)	22 (1.7%)	26 (2.7%)
**Donor: Recipient Weight Ratio < 0.8**	43 (4.4%)	49 (3.9%)	36 (3.7%)
**Donor: Recipient BSA Ratio**	1.17 [1.00, 1.38]	1.17 [1.02, 1.38]	1.15 [1.02, 1.33]
**Donor and Recipient BSA Differ by >20% in Either Direction**	467 (48.0%)	588 (46.4%)	431 (44.4%)
**Donor and Recipient Sex Differ**	439 (45.1%)	623 (49.1%)	452 (46.6%)
**Female Donor and Male Recipient**	194 (19.9%)	289 (22.8%)	199 (20.5%)
**Male Donor and Female Recipient**	245 (25.2%)	334 (26.3%)	253 (26.1%)

Data are expressed as number (percent) or median (interquartile range). LV (left ventricle), BSA (body surface area)

© 2020 Singh TP et al. *JAMA Network Open.*
